# The Rapid Effect of Bisphenol-A on Long-Term Potentiation in Hippocampus Involves Estrogen Receptors and ERK Activation

**DOI:** 10.1155/2017/5196958

**Published:** 2017-01-31

**Authors:** Xiaowei Chen, Yu Wang, Fang Xu, Xiaofei Wei, Junfang Zhang, Chuang Wang, Hua Wei, Shujun Xu, Peiyun Yan, Wenhua Zhou, Istvan Mody, Xiaohong Xu, Qinwen Wang

**Affiliations:** ^1^Ningbo Key Laboratory of Behavioral Neuroscience, Zhejiang Provincial Key Laboratory of Pathophysiology, School of Medicine, Ningbo University, Ningbo 315211, China; ^2^Chemistry and Life Sciences College, Xingzhi College, Zhejiang Provincial Key Laboratory of Ecology, Zhejiang Normal University, Jinhua 321004, China

## Abstract

Bisphenol-A (BPA), a widely used synthetic compound in plastics, disrupts endocrine function and interferes with physiological actions of endogenous gonadal hormones. Chronic effects of BPA on reproductive function, learning and memory, brain structure, and social behavior have been intensively investigated. However, less is known about the influence of BPA on long-term potentiation (LTP), one of the major cellular mechanisms that underlie learning and memory. In the present study, for the first time we investigated the effect of different doses of BPA on hippocampal LTP in rat brain slices. We found a biphasic effect of BPA on LTP in the dentate gyrus: exposure to BPA at a low dose (100 nM) enhanced LTP and exposure to BPA at a high dose (1000 nM) inhibited LTP compared with vehicle controls. The rapid facilitatory effect of low-dose BPA on hippocampal LTP required membrane-associated estrogen receptor (ER) and involved activation of the extracellular signal-regulated kinase (ERK) signaling pathway. Coadministration of 17*β*-estradiol (E_2_, the primary estrogen hormone) and BPA (100 nM) abolished both the BPA-induced enhancement of LTP and the E_2_-induced enhancement of baseline fEPSP, suggesting a complex interaction between BPA- and E_2_-mediated signaling pathways. Our investigation implies that even nanomolar levels of endocrine disrupters (e.g., BPA) can induce significant effects on hippocampal LTP.

## 1. Introduction

Bisphenol-A (BPA) is a widely used synthetic compound included in polycarbonate plastics and epoxy resins, for example, in food and beverage containers, dental prostheses, compact discs, and baby bottles. It is capable of acting as an endocrine disrupter and interferes with actions of endogenous gonadal hormones (e.g., estrogen or androgen) at low concentrations. BPA can bind to estrogen receptors (ERs) at low concentration and thus affects normal hormonal regulation and endocrine function [1]. A large number of studies have indicated that chronic exposure to the low-dose (nanomolar) BPA during fetal/neonatal stages inhibits sexual differentiation and nonreproductive behaviors of adult animals [2–4].

Although the widespread effects of BPA on reproductive function, brain structure, and social behavior have been investigated, recent studies reported controversial actions of BPA on learning and memory, ranging from deficits to no effect and to enhancements. In rodents, pre- and perinatal exposures to BPA at or below the TDI (tolerable daily intake; ⩽50 *μ*g/kg/day) have resulted in adverse effects on memory processes [4–9]. Adolescent exposure to BPA below the TDI impairs spatial memory in rats [5]. In contrast, other studies have shown that chronic oral exposure to BPA does not alter memory processes of adult male or ovariectomized (OVX) female rats [10, 11]. We previously found that acute exposure to BPA rapidly enhanced short-term passive avoidance memory in the developing rats [12]. The underlying mechanism is unclear. The role of BPA in synaptic remodeling in brain areas involved in learning and memory is also controversial. Adolescent exposure to low-dose BPA inhibited spinogenesis and synaptic modification in hippocampi of rodents [13]. BPA inhibited 17*β*-estradiol (E_2_)-induced formation of dendritic spine synapses in hippocampal CA1 area and prefrontal cortex of adult ovariectomized rats or nonhuman primates [14, 15]. However, other studies have shown the facilitatory effects of BPA on synaptic plasticity in neuronal development. Exposure to BPA at low doses (<100 nM) enhanced both dendritic and synaptic development in cultured hypothalamic cells [16, 17]. Exposure to BPA at 10–100 nM for 30 min rapidly increased the spine density dendritic filopodia mobility of the hippocampus [18]. Nanomolar doses of BPA rapidly modulated spinogenesis in adult hippocampal neurons [19]. Our previous study has also identified the facilitatory effect of BPA on dendritic morphogenesis of cultured hippocampal neurons through ER activation [12].

The long-lasting plasticity of synaptic transmission, as long-term potentiation (LTP) or long-term depression (LTD), is thought to be the cellular basis of learning and memory processes. Interestingly, it has been reported that exposure to BPA at low concentrations (10–100 nM) rapidly enhanced LTD in CA1 and CA3 but suppressed LTD in the dentate gyrus of the hippocampus [20, 21]. However, no studies have assessed the potential of BPA to influence LTP, and the underlying mechanisms are yet largely unknown.

The extracellular signal-regulated kinase (ERK) signal pathway is a component of a mitogen-activated protein kinase (MAPK) signaling cascade which regulates a variety of important cellular events. Recently, evidence highlights the ERK-mediated effects of estrogen and xenoestrogens in the brain [22]. Our previous studies have demonstrated that ERK signaling is involved not only in the chronic effect of BPA on dendritic morphogenesis in hippocampal neurons but also in the rapid effect of BPA on passive avoidance memory of young rats [12, 23].

In the present study, we investigated the dose-dependent effect of BPA on hippocampal LTP and explored the downstream intracellular pathways. In addition, we examined the synergistic role of BPA and E_2_ in hippocampal LTP. Therefore our study provides additional information on possible mechanisms for the effects of BPA on synaptic plasticity in brains.

## 2. Materials and Methods 

### 2.1. Animal and Drug Treatment

All experiments were carried out on male Wistar rats (Weight 120–140 g, age 5-6 weeks). The use of animals for experimental procedures was carried out in accordance with Guidelines for the Care and Use of the Laboratory Animals of Ningbo University, China.

### 2.2. Preparation of Slices

All experiments were conducted on transverse slices of the rat hippocampus. The brains were rapidly removed after decapitation and placed in cold oxygenated (95% O_2_, 5% CO_2_) artificial cerebral spinal fluid (ACSF). Slices were cut at a sickness of 350 *μ*m using a VT 1000S vibroslicer (Leica, Germany) and placed in a storage chamber containing oxygenated medium at room temperature (20–22°C) for 1 h. The slices were then transferred to a recording chamber and continuously superfused at a rate of 5-6 mL/min at 30–32°C. The ACSF contained (mM) NaCl, 120; KCl 2.5, NaH_2_PO_4_, 1.25; NaHCO_3_ 26; MgSO_4_, 2.0; CaCl_2_, 2.0; d-glucose 10. All solutions contained 100 *μ*M picrotoxin (Sigma, St Louis, MO, USA) to block GABA_a_-mediated activity.

### 2.3. In Vitro Electrophysiological Techniques

The electrophysiological techniques were applied according to our previous reports [24, 25]. Presynaptic stimulation was applied to the medial perforant pathway of the dentate gyrus using a bipolar insulated tungsten wire electrode, and field excitatory postsynaptic potentials (fEPSPs) were recorded at a control test frequency of 0.033 Hz from the middle one-third of the molecular layer of the dentate gyrus with a glass microelectrode. The inner blade of the dentate gyrus was used in all studies. In each experiment, an input-output curve (afferent stimulus intensity versus fEPSP amplitude) was plotted at the test frequency. For all experiments, the amplitude of the test EPSP was adjusted to one-third of maximum (~1.2 mV). LTP was evoked by high-frequency stimulation (HFS) consisting of two trains (each of two stimuli at 100 Hz for 1 s, intertrain interval 15 s) with the stimulation voltage increased during the HFS so as to evoke an initial EPSP of the train of double the normal test EPSP amplitude.

### 2.4. Statistics

Recordings were analyzed using pCLAMP 10.3 software (Axon Instruments, Foster City, CA, USA). Values are the means ± SEM for *n* slices. All brain slices in the same group were from different animals. In most experiments, the amplitude of fEPSPs measured 40 min after HFS (post-HFS) was shown, unless indicated otherwise. Two-tailed Student's *t*-test and one-way ANOVA were used for the detailed statistical analysis where appropriate; *p* < 0.05 was considered statistically significant.

### 2.5. Agents

All drugs were applied through the perfusion medium. BPA was purchased from Shanghai Chemical Reagent Research Institute (Shanghai, China). 17*β*-E_2_ and U0126 were purchased from Cell Signaling (Boston, MA, USA). ICI182,780 was purchased from Tocris (Ballwin, MO, USA). All reagents were dissolved in dimethyl sulphoxide (DMSO, from Sigma, St. Louis, MO, USA) and then diluted in ACSF (0.05% vehicle). Control levels of LTP were measured on slices perfused with vehicle (DMSO) alone.

## 3. Results

### 3.1. The Facilitatory Effect of Low-Dose BPA on LTP in the Dentate Gyrus

**Figure 1:**
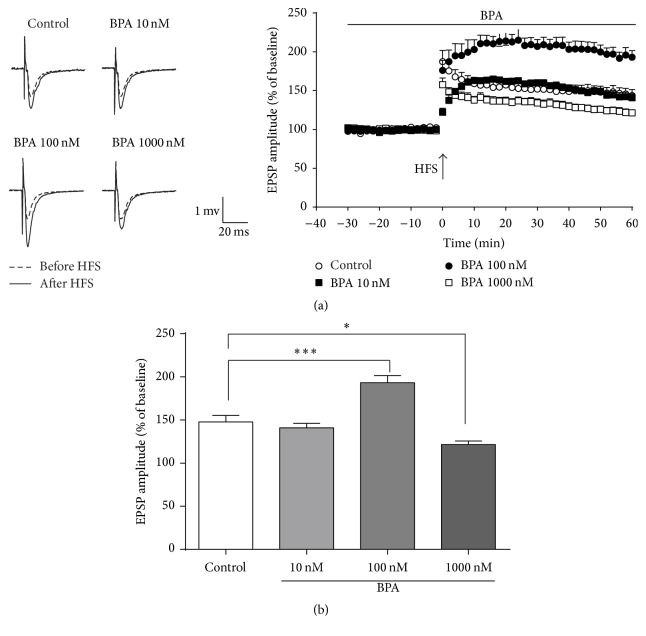
The biphasic effect of BPA on LTP in rat dentate gyrus in vitro. (a) High-frequency stimulation induced LTP in the medial perforant path of the dentate gyrus of acute rat hippocampus slices (open circles, *n* = 8). Applications of BPA are indicated at concentrations of 10 nM (filled squares, *n* = 8), 100 nM (filled circles, *n* = 8), and 1000 nM (open squares, *n* = 8), respectively. All hippocampal slices were preperfused with ACSF, 30 min before HFS, to obtain baseline EPSP amplitude. (b) Summary of the major experimental outcomes. The average fEPSP amplitudes at 60 min after HFS in separate perfusion of different concentration BPA. Applications of BPA 100 nM and BPA 1000 nM have significant effects on LTP, ^*∗*^*p* < 0.05, ^*∗∗∗*^*p* < 0.001 as compared to controls. Solid and dashed example traces before HFS and after HFS, respectively.

We first investigated the dose-dependent effect of BPA (10, 100, and 1000 nM; added to the ASCF 60 min before HFS) on synaptic plasticity of perforant path-granule cell synapses induced by HFS in the dentate gyrus (DG). We found that application of 10 nM BPA did not have any effect on LTP (140.8 ± 5.2% of baseline, *n* = 8) compared with vehicle controls (143.7 ± 7.6% of baseline, *n* = 8, *p* > 0.05, Figures 1(a) and 1(b)). However, 100 nM BPA increased LTP (193.1 ± 8.3% of baseline, *n* = 8) compared to control (143.7 ± 7.6% of baseline, *n* = 8, *p* < 0.001, Figures 1(a) and 1(b)). In contrast, application of BPA 1000 nM resulted in an inhibition of LTP in DG (121.1 ± 4.0% of baseline, *n* = 8, *p* < 0.05, Figure 1(b)), indicating a biphasic effect of low-dose (100 nM) and high-dose (1000 nM) BPA on hippocampal LTP.

### 3.2. The BPA-Enhanced LTP Requires Activation of ERs

**Figure 2:**
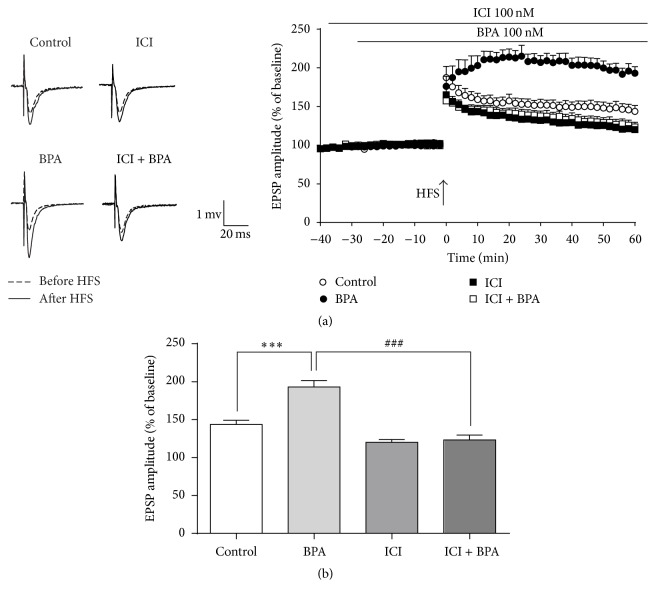
The enhancement of BPA on hippocampal LTP was ER-dependent. (a) Administration of ICI 182,780 10 nM (an antagonist of ERs, filled square, *n* = 8) remarkably decreased the 100 nM BPA-induced enhancement of LTP. Pretreatment with the ERs antagonist ICI 182,780 30 min before BPA 100 nM (open squares, *n* = 8) application completely blocked BPA-enhanced LTP compared with BPA alone. (b) Figure columns express the average fEPSP amplitudes after HFS in separate perfusion or coperfusion of BPA 100 nM and ICI 182,780 100 nM, ^*∗∗∗*^*p* < 0.001 as compared to the control, ^###^*p* < 0.001 as compared to the BPA 100 nM. Solid and dashed example traces before HFS and after HFS, respectively.

To examine whether the enhancement of LTP by 100 nM BPA involves ERs, we add a high-affinity nonselective ER antagonist ICI 182,780 (100 nM) into bath solution 30 min before BPA application. Application of ICI 182,780 had no effect on LTP (120.6 ± 3.7% of baseline, *n* = 8, controls: 140.8 ± 5.2% of baseline, *n* = 8. *p* > 0.05, Figure 2(b)) but blocked BPA-enhanced LTP (123.4 ± 6.2% of baseline, *n* = 8, *p* < 0.001, Figure 2(b)), suggesting that the facilitatory effect of BPA (100 nM) on LTP in hippocampal dentate gyrus requires the activation of ERs.

### 3.3. BPA-Enhanced LTP Involves ERKs

**Figure 3:**
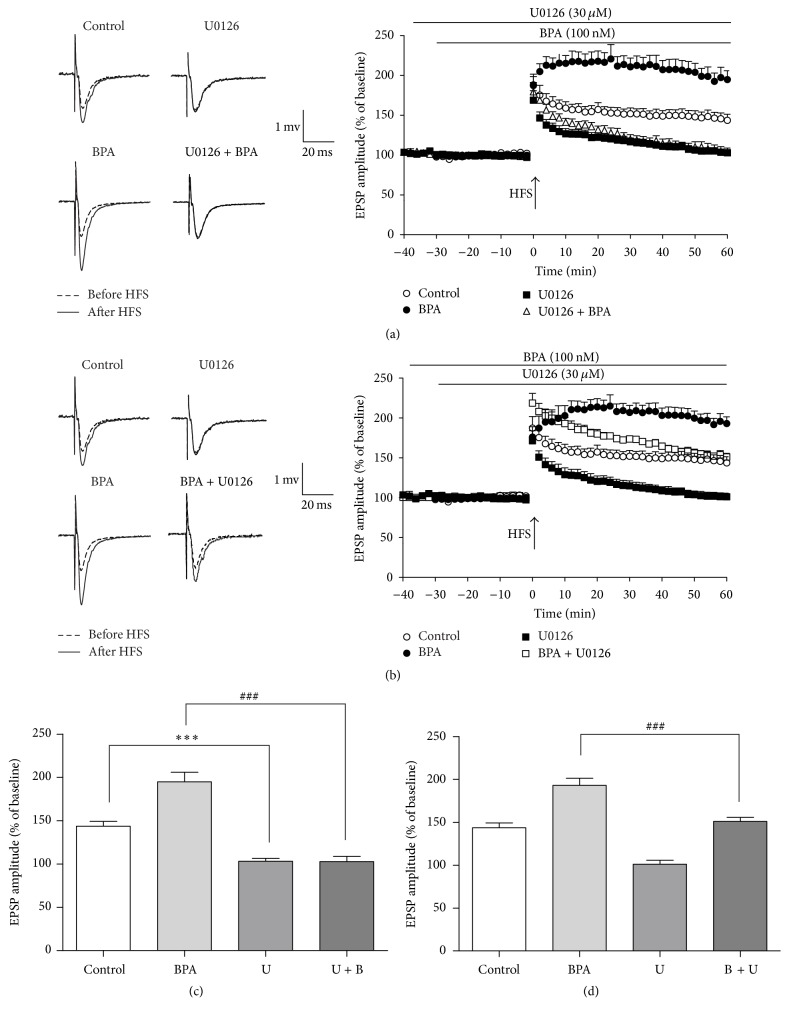
ERK signal pathway was involved in BPA-enhanced LTP. Pretreatment with ERK inhibitor U0126 for 30 min before BPA 100 nM (open triangles, *n* = 8) completely blocked LTP compared with controls. (b) Pretreatment with BPA 100 nM 30 min before the ERK inhibitor (open squares, *n* = 8) application remarkably decreased the BPA effect as compared with BPA 100 nM alone (open squares). (c, d) Figure columns showing the average fEPSP amplitudes at 60 min after HFS in separate perfusion or coperfusion of BPA 100 nM and U0 126 30 *μ*M, ^*∗∗∗*^*p* < 0.001 as compared to the control, ^###^*p* < 0.001 as compared to the BPA 100 nM. Solid and dashed example traces before HFS and after HFS, respectively.

To explore the downstream signaling pathway of the BPA-enhanced LTP in rat hippocampus, we examined whether the ERK pathway is involved. Application of 100 nM U0126 (a MEK1/2 or ERK inhibitor) 60 min before HFS did not alter the baseline fEPSP but inhibited the hippocampus LTP in rat dentate gyrus compared with vehicle controls (103.1 ± 3.5% of baseline, *n* = 8, *p* < 0.001, Figures 3(a) and 3(c)). In addition, pretreatment of 100 nM U0126 added 30 min before BPA application completely blocked BPA-enhanced LTP (102.8 ± 6.1% of baseline, *n* = 8, *p* < 0.001, Figure 3(c)). However, pretreatment of BPA (added 30 min before U0126 application) resulted in partial inhibition of BPA-enhanced LTP (151.0 ± 4.7% of baseline, *n* = 8, *p* < 0.001, Figure 3(d)). These results indicate that activation of ERK pathway is not only required for physiological LTP but also necessary for the facilitatory effect of BPA on LTP in the dentate gyrus.

### 3.4. The Effects of BPA and E_2_ on Baseline fEPSP and LTP Enhancement

**Figure 4:**
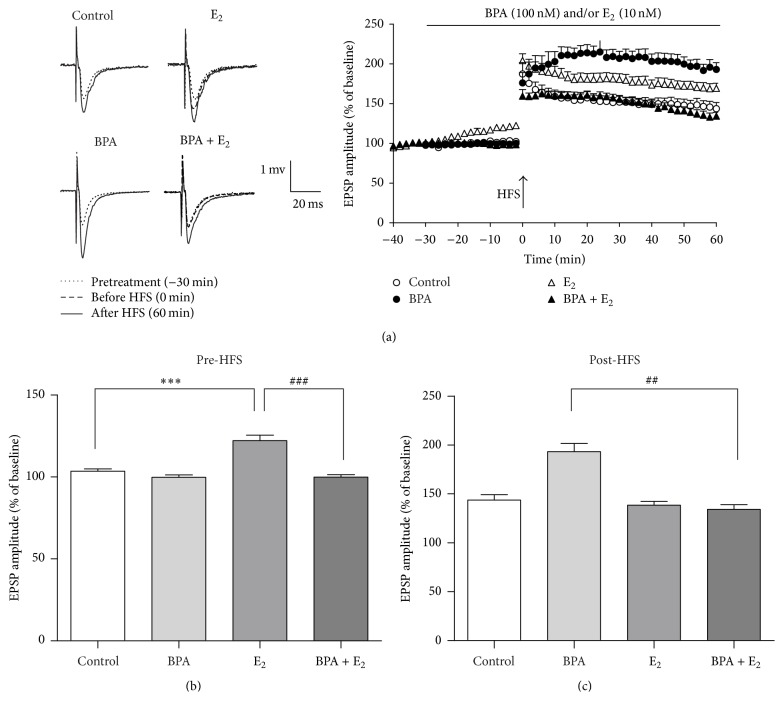
The enhancement of BPA on hippocampal LTP was abolished by E_2_ treatment. Enhanced LTP by E_2_ (10 nM) in hippocampal area DG (open triangles, *n* = 8). Coadministration of BPA 100 nM with E_2_ (filled triangles, *n* = 8) had no effect on LTP compared with untreated controls. (b) Pretreatment by E_2 _enhanced the pre-HFS EPSP amplitudes, ^*∗∗∗*^*p* < 0.001 as compared to controls, ^###^*p* < 0.001 as compared to the E_2_ 10 nM. (c) Comparison of different groups the fEPSP amplitudes at 60 min. E_2_ had no effect on LTP compared with untreated controls without baseline increase in EPSP amplitude (before the HFS). ^*∗∗∗*^*p* < 0.001 as compared to the control, ^##^*p* < 0.01 as compared to the BPA 100 nM. Solid and dashed traces are examples of before treatment, after treatment, and after HFS, respectively.

Previous studies have reported the facilitatory effect of E_2_ on both baseline fEPSP and LTP induction [26]. Here, we applied 10 nM E_2_ on rat brain slice and observed a significant increase (~20–30%) of baseline fEPSP compared with vehicle controls (Figures 4(a) and 4(b)). However, coapplication of BPA (100 nM) and E_2_ reversed the enhancement of baseline fEPSP induced by E_2_ (Figure 4(b)). In terms of LTP enhancement, E_2_ treatment did not enhance LTP while comparing fEPSP before HFS (at 0 min) and after HFS (at 60 min) in the dentate gyrus (Figures 4(b) and 4(c)). Unexceptionally, coapplication of E_2_ and BPA blocked both the BPA-induced enhancement of LTP and the E_2_-induced enhancement of baseline fEPSP (Figures 4(b) and 4(c)).

## 4. Discussion

### 4.1. The Rapid Facilitatory Effect of Low-Dose BPA on Hippocampal LTP Is ER-Dependent and Involves Activation of ERK Pathway

The rapid effect of BPA on synaptic plasticity has been investigated by several studies. It is shown that low-dose BPA (10 nM) increases Ca influx, enhances filopodia flexibility in cultured hippocampal neurons, and rapidly modulates spinogenesis in adult hippocampal slices [18, 19]. These effects have been reported to relate to ERs and MAPK activation [19]. In the terms of memory-related synaptic plasticity (e.g., LTP and LTD), the effect of BPA has been less investigated. Hasegawa et al. [21] have reported the BPA-induced enhancement of LTD in CA1 region of rat hippocampus, but this effect does not require ER activation [21]. However, we here demonstrate that low-dose BPA (100 nM) significantly enhances LTP in rat DG region and this facilitatory effect of BPA on LTP depends on ER activation since E_2_ antagonist ICI 182,780 completely abolishes the BPA enhancement on LTP.

There are two types of ERs: one type is nuclear estrogen receptors (nERs), which are members of the nuclear receptor family of intracellular receptors, including ER*α* and ER; the other type is membrane estrogen receptors (mERs), which are mostly G protein-coupled receptors, including Gq-coupled mER (Gq-ER), GPER1 (formerly GPR30), and ER-X [27]. In the genomic mechanism, E_2_ binds to ER*α* and ER*β* in the cytoplasm, and then the E_2_-ER complex translocates into the nucleus, binds to an estrogen response element on the DNA, and finally facilitates gene transcription. The nongenomic mechanism involves actions of mERs at the plasma membrane: ER*α* and ER*β* interact with mERs to rapidly activate extracellular signal-regulated kinase (ERK) cell signaling, which further triggers epigenetic processes, gene expression, and other cell signaling pathways [22]. Although it is not clear which type of ER(s) is involved in the facilitatory effect of BPA on LTP because ICI 182,780 blocks both nERs and mERs, the rapid effect of BPA (within 1 h) indicates a greater contribution of the nongenomic mER signaling to the BPA-induced enhancement of hippocampus LTP. Considering the essential roles of glutamate receptors (AMPA, NMDA, and metabotropic glutamate receptor) in the hippocampal LTP and the interactions between glutamate receptors and mERs, the glutamate receptors may also be involved in BPA-induced enhancement of LTP.

Growing evidence demonstrates that the hippocampal ERK signaling is necessary for E_2_ to enhance hippocampal memory consolidation [28, 29]. Here our results confirm that ERK activation is also required for the BPA-induced enhancement of LTP. It is interesting that the blockade of ERK pathway did not completely inhibit BPA-enhanced LTP while the slices were preincubated with BPA for 30 min before U0126 treatment. The reason may be due to the rapid effect of BPA on LTP since preincubation of BPA may already launch certain rapid downstream effects to enhance EPSP amplitude after high-frequency stimulation, whereas some slow effects of BPA requiring ERK activation are inhibited by following the application of U0126. These results are consistent with our previous findings on cultured rat hippocampal neurons that exposure to BPA for 30 min rapidly enhances the motility and the density of dendritic filopodia through the ER-mediated pathway [30]. Finally, our results also show that high-dose BPA (1000 nM) could severely inhibit hippocampal LTP, indicating a complex mechanism of BPA actions on neuroplasticity in hippocampi.

### 4.2. BPA and E_2_ Differently Influence Hippocampal LTP and There Might Be a Complex Interaction between Them

Estrogen (e.g., E_2_) is also locally synthesized within the hippocampus in addition to the gonads. Mounting articles demonstrate that E_2_ influences hippocampal memory [31, 32]. A number of studies have reported rapid effects of E_2_ on LTP, LTD, and spinogenesis in the hippocampus. Low concertation of E_2_ (1 nM) rapidly enhances LTD in CA1, CA3, and dentate gyrus of the hippocampus. The density of thin type spines increases in CA1 pyramidal neurons within 2 h after application of 1 nm estradiol and this enhancement of spinogenesis requires ERs and MAPK signals [33]. Vedder et al. have demonstrated that E_2_-induced enhancements in both spatial memory and LTP occur within a similar time frame, linking E_2_-induced changes in LTP with hippocampal memory formation [34]. Our previous study also confirms that E_2_ (10 nM) significantly increases the total dendritic length and enhances motility and density of dendritic filopodia in cultured hippocampal neurons [12]. In the terms of hippocampal LTP, although application of E_2_ (1–10 nM) does not directly enhance LTP, it induces a baseline increase of the excitatory postsynaptic potential (EPSP) in CA1 neurons [26, 35].

Consistently, in the present study, we have shown a significant increase (~20–30%) of baseline fEPSP induced by application of E_2_. Molecular mechanisms of modulation through synaptic estrogen receptor (ER) and its downstream signaling are still unknown. It may involve a complex kinase network based on a recent study investigating the induction of LTP by the presence of E_2_ upon weak theta burst stimulation (a subthreshold stimulation that did not induce full-LTP) in CA1 region of the adult male hippocampus [36]. This E_2_-induced LTP is ER-dependent and requires activation of multiple kinases including ERK, protein kinase A (PKA), protein kinase C (PKC), phosphatidylinositol 3-kinase (PI3K), and calcium calmodulin kinase II (CaMKII) [36].

It is worth noting that although exposure to either low-dose BPA or E_2_ alone enhances LTP suppression of these effects is observed when low-dose BPA and low-dose E_2_ are administrated together. Our findings are consistent with a previous in vivo study showing that the E_2_-induced increase in synapse density is inhibited by the simultaneous application of BPA (40 ug/kg) and E_2_ (60 ug/kg) in ovariectomized rats for 30 min [37]. The underlying mechanism is still unclear and requires further exploration. A possible explanation might be the influence of the allosteric effect of BPA on ERs. Binding of BPA to ERs may change the structure of E_2_ binding sites and affect the affinity of E_2_ to ERs. However, recent studies highlight another possibility that fluctuations of local E_2_ levels during a learning event may be a key factor in learning and memory [32]. A study in adult nonhuman primates reported that elevated E_2_ level by applying exogenous E_2_ interferes with a cognitive function on the delayed response task in female monkeys [38]. Nevertheless, a study in finches has found that dynamic suppression of E_2_ synthesis during a learning event may be a critical component of learning processes [39]. Possibly, low-dose BPA alone may act as the ER modulator and has estrogen-like effects on synaptic plasticity in the hippocampus, whereas high-dose BPA alone may act as the ER disrupter and impair hippocampal LTP, LTD, and spinogenesis. However, in physiological states, if we take into account the locally synthesized E_2_ in the hippocampus and the importance of fluctuations of local E_2_ levels in cognitive circuits, a small amount of BPA could disturb the subtle regulation of E_2_ level and then influence hippocampal LTP.

## 5. Conclusions

In summary, we demonstrated biphasic effects of BPA on LTP in DG region of rat hippocampus: exposure to BPA at a low dose (100 nM) enhances LTP while to a high dose BPA (1000 nM) inhibits LTP. The rapid facilitatory effect of low-dose BPA on hippocampal LTP requires membrane-associated ER and involves activation of ERK signaling pathway. Coadministration of E_2_ and BPA (100 nM) abolishes BPA-induced enhancement of LTP and E_2_-induced enhancement of baseline fEPSP, suggesting a complex interaction between BPA- and E_2_-mediated downstream pathways. Our investigation about hippocampal LTP implies that even nanomolar low doses of endocrine disrupters (e.g., BPA) could induce significant effects on hippocampal synaptic plasticity.
